# Elongating Effect of the Peptide AEDL on the Root of *Nicotiana tabacum* under Salinity

**DOI:** 10.3390/plants11101352

**Published:** 2022-05-19

**Authors:** Larisa I. Fedoreyeva, Ekaterina N. Baranova, Inn A. Chaban, Tatyana A. Dilovarova, Boris F. Vanyushin, Neonila V. Kononenko

**Affiliations:** 1 All-Russia Research Institute of Agricultural Biotechnology, Timiryazevskaya 42, 127550 Moscow, Russia; greenpro2007@rambler.ru (E.N.B.); chaban@rambler.ru (I.A.C.); dilovarova@yandex.ru (T.A.D.); vanyush@belozersky.msu.ru (B.F.V.); nilva@mail.ru (N.V.K.); 2 N.V. Tsitsin Main Botanical Garden of Russian Academy of Sciences, Botanicheskaya 4, 127276 Moscow, Russia; 3 A.N. Belozersky Institute of Physico-Chemical Biology, Lomonosov Moscow State University, Leninskie Gory 1, 119991 Moscow, Russia

**Keywords:** root, elongation, *Nicotiana tabacum*, peptide AEDL, ROS, chromatin structure

## Abstract

The overall survival of a plant depends on the development, growth, and functioning of the roots. Root development and growth are not only genetically programmed but are constantly influenced by environmental factors, with the roots adapting to such changes. The peptide AEDL (alanine–glutamine acid–asparagine acid–leucine) at a concentration of 10^−7^ M had an elongating effect on the root cells of *Nicotiana tabacum* seedlings. The action of this peptide at such a low concentration is similar to that of peptide phytohormones. In the presence of 150 mM NaCl, a strong distortion in the development and architecture of the tobacco roots was observed. However, the combined presence of AEDL and NaCl resulted in normal root development. In the presence of AEDL, reactive oxygen species (ROS) were detected in the elongation and root hair zones of the roots. The ROS marker fluorescence intensity in plant cells grown with AEDL was much lower than that of plant cells grown without the peptide. Thus, AEDL protected the root tissue from damage by oxidative stress caused by the toxic effects of NaCl. Localization and accumulation of AEDL at the root were tissue-specific. Fluorescence microscopy showed that FITC-AEDL predominantly localized in the zones of elongation and root hairs, with insignificant localization in the meristem zone. AEDL induced a change in the structural organization of chromatin. Structural changes in chromatin caused significant changes in the expression of numerous genes associated with the development and differentiation of the root system. In the roots of tobacco seedlings grown in the presence of AEDL, the expression of *WOX* family genes decreased, and differentiation of stem cells increased, which led to root elongation. However, in the presence of NaCl, elongation of the tobacco root occurred via a different mechanism involving genes of the expansin family that weaken the cell wall in the elongation zone. Root elongation of plants is of fundamental importance in biology and is especially relevant to crop production as it can affect crop yields.

## 1. Introduction

Roots are typically the underground organ of plants and not only provide structural support for the aboveground part of the plant but also supply and accumulate nutrients and water that are vital for plant development [1]. The roots contain a number of hormones that control all life-supporting processes of development, not only the roots but also the whole organism [1,2]. The overall survival of a plant depends on the roots’ development, growth, and functioning. Root development is challenging to study. For dicotyledonous plants, the simple taproot architecture predominantly consists of an embryonically derived primary root with lateral roots that emerge post-embryonically. For plants with a fibrous root system, such as the monocots, the complexity of the root architecture is increased by the presence of additional root types, including various adventitious and lateral roots. Root development and growth are not only genetically programmed but are constantly influenced by environmental factors. Roots perceive various signals from the environment and process these signals to reprogram their development and adapt to changes in the environment. Flexibility in the root system is essential for survival [2,3].

The production of new root cells from stem cells, which are known as initials, occurs in the root apical meristem (RAM), with the cells going through three developmental phases on the way to maturity [1]. In the meristematic zone (MZ), the cells divide several times, forming a pool of cells that will elongate and differentiate. In the elongation zone (EZ), cells lose their ability to divide and grow in length many times their width. Finally, in the zone of differentiation (DZ), cells acquire their specialized characteristics and functions. The quiescent center (QC) is required for the specification of the stem cell niche and maintenance of the undifferentiated state of the stem cell initials [4]. 

The regulation of root growth is a complex process controlled by intricate networks of gene interactions both in time and in space. Genes associated with the development of the root system were studied through gene expression profiling of the *Arabidopsis* root [5] and sequencing of the *Arabidopsis* root transcriptome [6]. The *WOX* genes (Wuschel-related homeobox *WUS*) form a family of plant-specific homeodomain transcription factors, whose members play an important role in developing a wide range of processes [7]. The *WOX* genes play crucial roles in plant growth and development, including regulation of stem cells [8], embryo patterning [9], and flower development [10]. Proteins in this superfamily commonly contain a homeobox domain comprising 60–66 amino acids folded into a helix-turn-helix structure, which can be recognized by specific DNA to regulate the expression of a target gene at a certain point [7,11]. The *WOX* genes were first identified as determining the fate of cells during embryonic development, as well as being instrumental in maintaining stem cell niches in plants [4]. Overexpression of *WOX1* results in the formation of an abnormal meristem in *A. thaliana* [12]. 

*WOX2* and *WOX8* are expressed in the zygote and then confined to apical and basal cells, respectively, and play an essential role in patterning early embryos [13,14]. *WOX4* participates in the regulation of lateral root formation in plants [15], while *WOX5* is an essential regulator for the formation of the correct root pattern of the root apical meristem [16]. *WOX7*, which is expressed in lateral roots, inhibits lateral root development in a sugar-dependent manner [17]. *WOX11* and its homolog *WOX12* are involved in de novo root organogenesis [18]. The QC regulates the size of the stem cell pool by limiting the expression of WOX5 [19,20]. Peptide CLE40 (CLAVATA3-CLV3) acts through receptor kinase ACR4 to exclude *WOX5* expression outside the QC [21]. Other regulators of stem cell identity include small, secreted peptides known as root meristem growth factors, which contribute to postembryonic maintenance of the stem cell niche [22].

Plant growth and development must transcend the boundaries of the plant cell wall, and regulation of cell-wall extensibility is a necessary process accompanying cell expansion and division. In fast-growing tissues such as roots, the weakening of the cell wall is a highly regulated process and is coordinated with development. Controlled cell expansion allows plants to adapt to different environmental conditions. The rigid cell wall can stretch under the action of turgor pressure, changing its shape and size [23,24]. Hence, turgor pressure is thought to stimulate plant growth. Tissues harden when turgor pressure rises and can soften or even wilt when turgor falls. The growth and elongation of the plant cell depend on the loosening of the rigid polysaccharide structure of the cell wall. Molecular modifications of the polysaccharides are responsible for the loosening as they weaken the cross-links. There are four recognized molecular mechanisms of cell-wall loosening that involve degrading agents, including expansins [25], endotransglucosylase xyloglucan/hydrolases (XTHs) [26], endo (1,4) -β-D-glucanases [27], and a hydroxyl radical (• OH) [28].

Cellulose is the main component of the plant cell wall and is a polysaccharide of glucose units. Plants contain a family of secreted endo (1,4)-β-D-glucanases (also called “cellulases”) [29], which hydrolyze the β-glycosidic bond between glucose residues [30]. The hydroxyl radical (• OH) is a powerful form of reactive oxygen species (ROS) that has an important role in signaling and cell death. The formation of • OH in the cell wall can weaken the wall due to the destruction of hydrogen bonds by non-enzymatic binding of polysaccharides to the hydrogen atom [31]. In addition to cellulose, pectin is a major component of the plant cell wall. Enzymes associated with pectin, such as pectin lyase, pectin esterase/pectin methylesterase (PME), pectin methylesterase inhibitor (PMEI) family protein, and pectinase, have crucial functions in weakening the cell wall [32]. Expansins are pH-dependent proteins that attenuate intercellular interactions and are involved in cell expansion, as well as various developmental processes that require cell-wall modification. The expansins superfamily comprises four families: α-expansin (*EXPA*), β-expansin (*EXPB*), expansin-like A *(EXLA*), and expansin-like B (*EXLB*). It has been experimentally proven that only the EXPA and EXPB proteins can cause a weakening of the cell wall [33,34]. Altering the expression of one expansin gene (*EXPA5*) led to a marked change in root length caused by elongation of the cells and not by an increase in cell numbers. This suggested that expansin proteins significantly impact cell-wall loosening and elasticity, which are important processes of root development.

The weakening of the cell wall is regulated by many factors, including hormones and environmental signals [28]. Abscisic acid (ABA), brassinosteroids, cytokinin, ethylene, gibberellic acid (GA), and auxin (IAA) plays the most important role in root development [35,36].

ROS also participate in the control of the transition from proliferation to differentiation [37]. The transcription factor UPBEAT1 (UPB1), expressed in the transition zone, is directly regulated by peroxidases, which alter the distribution of ROS. Cell proliferation requires high levels of O^2−^, while high levels of H_2_O_2_ induce differentiation. Ethylene is the predominant hormone produced by plants upon exposure to high salt concentrations [38,39,40]. In plant cells, ethylene is produced from the precursor 1-aminocyclopropane-1-carboxylic acid (ACC) [41]. Ethylene production induces three critical reactions, collectively known as the triple response, including inhibition of hypocotyl and root elongation, hypocotyl swelling, and increased apical hook tightness [42]. Stomatal closure affects abiotic stress in plants and is controlled by a complex signaling system that leads to increased stress resistance [43].

Previously, it was found that the exogenous short biologically active tetrapeptide AEDL (alanine–glutaminic acid–asparaginic acid–leucine) significantly influenced the growth, development, and differentiation of *Nicotiana tabacum* callus cultures. [44]. AEDL stimulates the formation and growth of leaves in plant regenerants. In tobacco cells, the peptide AEDL modulates the expression of genes responsible for tissue formation and cell differentiation.

The aim of the current investigation was to study the effect of the AEDL tetrapeptide on the root of *N. tabacum*, as well as the combined effect of AEDL and NaCl on the plants.

## 2. Results

### 2.1. Morphometric Data

**Figure 1:**
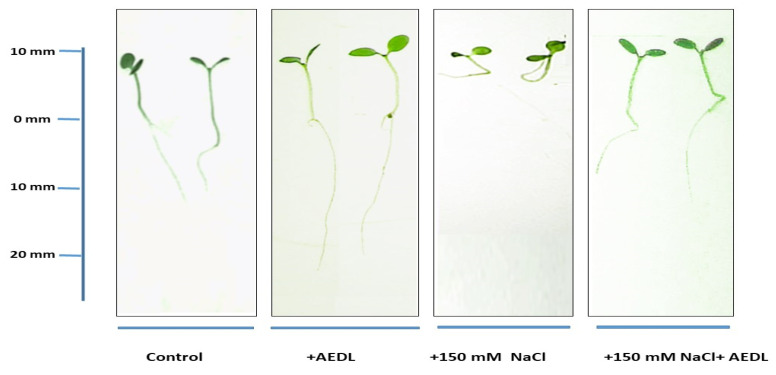
Twenty-eight days of tobacco seedling growth without and with additives.

Morphometric assessment of tobacco seedlings *Nicotiana tabacum* L. under the influence of various treatments was performed to investigate possible changes in plants during growth. Growing tobacco on standard MS medium in the presence of 10^−7^ M peptide AEDL resulted in a twofold increase in taproot length compared with the control (Figure 1, Table 1). In contrast, the height of the seedlings only increased by 20%. A visible symptom of the effects of salinization is the disturbance of plant growth. The addition of 150 mM NaCl to the nutrient medium caused a decrease in the growth of both the root system and aerial organs in the studied tobacco samples (Figure 1, Table 1). The difference in shoot height between tobacco samples grown under different conditions was 20%. According to biometric data, the root system of tobacco grown in the presence of NaCl and AEDL was more developed and less susceptible to the negative effects of chloride salinity compared with the plants grown in the presence of 150 mM NaCl alone.

**Figure 2:**
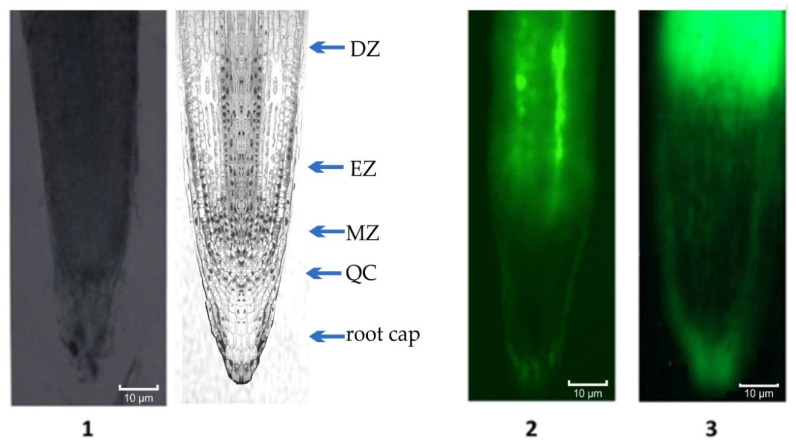
Visualization of FITC-labeled AEDL in tobacco roots. (**1**) Root incubated with FITC; (**2**) root incubated with FITC-AEDL; (**3**) root incubated with FITC-AEDL with 3× magnification of MZ. DZ—zone of differentiation; EZ—elongation zone; MZ—meristematic zone; QC—quiescent center.

Fluorescein isothiocyanate (FITC)-labeled AEDL was used to study the penetration of the peptide into tobacco root cells (Figure 2). Fluorescence was detected in the cell walls, cytoplasm, and nucleus after incubation of tobacco seedling roots in a FITC-AEDL solution for 20 h. However, the fluorescence intensity in different root cells was different (Figure 2). In the meristematic zone of the root, some cells were devoid of fluorescence. The f FITC–AEDL signal was observed in the apical zone of the root cap and in the epidermal cells. Concurrently, the roots of tobacco plants that were incubated with FITC exhibited no fluorescence (Figure 2(1)). Strong fluorescent signals were observed in the elongation zones and differentiation when roots were incubated with FITC-AEDL. In the meristem zone, the fluorescent signal was weak, and it was possible to visualize only after a significant increase of 3×. Thus, the emergence and accumulation of FITC-AEDL in the root is, to a certain extent, cell (tissue)-specific.

### 2.2. TEM Analysis

**Figure 3:**
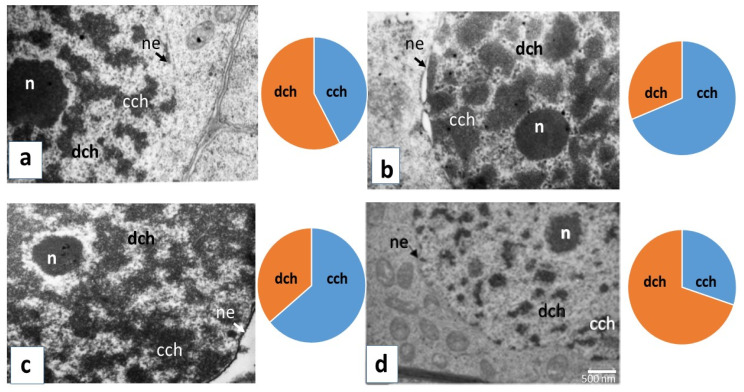
Nucleus ultrastructure in MZ of the main root cells of *Nicotiana tabacum* during germination in water (**a**). In the presence of AEDL (**b**), in the presence of 150 mM NaCl (**c**), and in the joint presence of NaCl and AEDL (**d**) after 28 days of germination. Symbol: n—nucleolus, ne—nuclear envelope, cch—condensed chromatin, dch—decondensed chromatin. Bar—500 nm.

Structural transformations of the plant cells’ nuclei during cultivation with AEDL and under the action of NaCl were studied by the method of electron microscopy. The location and relationship between condensed and decondensed chromatin were dependent on AEDL application and salinity (Figure 3). Treatment with the peptide AEDL altered the structural organization of nuclei, as evidenced by the increase of condensed chromatin square. 

In the control, clusters of condensed chromatin in the nucleoplasm were evenly distributed, decondensed chromatin and the usual number of ribosomes were detected, and the nucleolus was of a small and irregular shape. Salinity increases the proportion of condensed chromatin compared to the control (~27%); clear damage to the nuclear membrane was seen (Figure 3b, Table S1). The use of the peptide AEDL leads to a decrease in the amount of decondensed chromatin compared to the control (~22%) and an increase in condensed chromatin (~22%). After the addition of the peptide to the medium in the tobacco root cells, the condensed chromatin formed a network over the entire surface of the nucleus; the nucleolus had a free decondensed chromatin areola that is close to a round shape (Figure 3c). The electron micrographs showed changes in the electron density of the nucleus upon treatment of plant cells with NaCl. Under the combined action of salinity and AEDL, the area of condensed chromatin in the nucleus decreased in comparison with the use of only one peptide (~34%), while the decondensed chromatin increased (~34) (Figure 3d). Thus, the action of the peptide AEDL affects a change in the structural organization of the nuclei, a decrease in the amount of decondensed chromatin, and an increase in condensed chromatin, which is likely to cause significant changes in the expression of a number of genes associated with development and differentiation.

### 2.3. ROS Distribution

**Figure 4:**
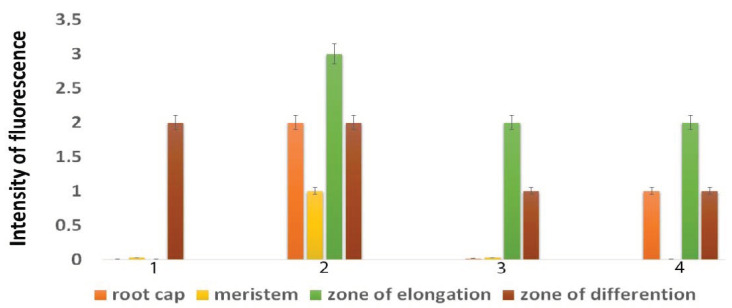
Intensity of fluorescence of ROS cells in the zones of 28 days tobacco roots. (1) Control; (2) in the presence AEDL; (3) in the presence 150 mM NaCl; (4) in the joint presence AEDL and 150 mM NaCl. Standard deviations are shown according to Student’s criterion, *p* < 0.05.

**Figure 5:**
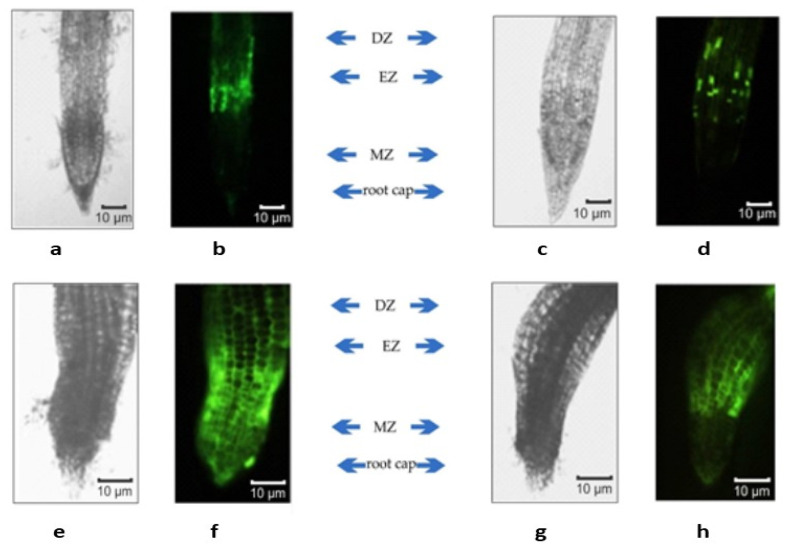
Distribution of ROS+ and ROS—cells in the zones of 28 days tobacco roots. (**a**,**b**) Control; (**c**,**d**) in the presence AEDL; (**e**,**f**) in the presence 150 mM NaCl; (**g**,**h**) in the joint presence AEDL and 150 mM NaCl. Bar—10 µm.

The staining of tobacco roots with a fluorescent dye for ROS revealed that ROS were found in many root tissues under NaCl treatment; however, the intensity of the staining varied in cells from different zones of the roots. Because not all of the root zones had the same fluorescence intensity, the distribution of cells with increased ROS levels was evaluated in different root zones (Figure 4). After NaCl treatment, the most intense fluorescence was observed in the root cap, meristematic, elongation, and root hair zones (Figure 5). In the control seedlings, ROS were present only in the root hair zone. After AEDL and NaCl treatment, intense fluorescence was absent in the meristematic zone but was detected in the root cap, elongation, and root hair zones of the seedling roots. In the AEDL variant, ROS were detected in the zones of elongation and root hair zones (Figure 4). Moreover, the intensity of Carboxy-H_2_DFFDA fluorescence ROS marker in plant cells grown with the addition of AEDL was much lower, both with and without salinity, compared with the control seedlings, indicating the protective properties of AEDL under oxidative stress.

### 2.4. Expression of EXPA3 and EXPA5 Genes and Genes of the WOX Family

**Figure 6:**
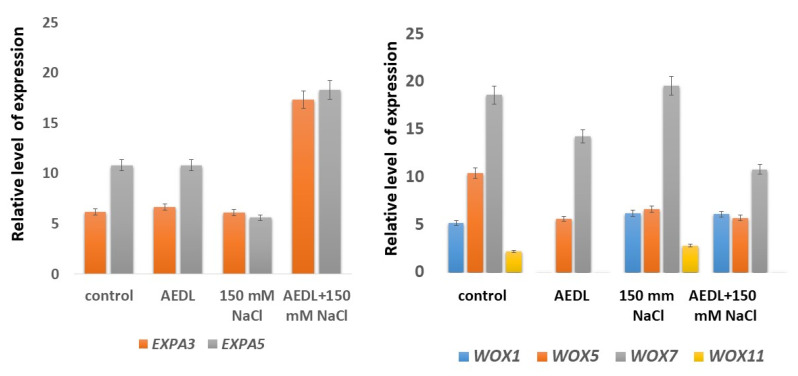
Relative expression levels of genes of *EXPA3* and *EXPA5* (**left**) and *WOX* family (**right**) in tobacco seedling roots. Standard deviations are shown according to Student’s criterion, *p* < 0.05.

The expression of the expansin genes *EXPA3* and *EXPA5* in the roots of tobacco grown in the presence of the peptide AEDL, NaCl alone, or AEDL and NaCl is shown in Figure 6. Relative expression of the *EXPA3* gene was lower than that of the *EXPA5* gene in both control tobacco seedlings and tobacco seedlings grown in the presence of the peptide AEDL. In the seedlings grown in the presence of 150 mM NaCl, as well as in the presence of salt and peptide AEDL was practically the same. The expression profiles of expansins *EXPA3* and *EXPA5* in the control seedlings and in the seedlings grown in the presence of AEDL were similar. This may indicate that the peptide AEDL does not affect the expression of the expansins and that the lengthening of the root in the presence of the peptide does not occur due to the weakening of the cell wall in the EZ. In the presence of a high concentration of NaCl, the expression of *EXPA5* was suppressed by 50%. This decrease in expansin expression was accompanied by a decrease in root length and root bending. However, in tobacco grown in the presence of NaCl and AEDL, there was a significant increase (more than threefold) in the expression of both *EXPA3* and *EXPA5*. As a result of the increased expression of the expansin genes, the roots of the seedlings were lengthened, and the curvature of the roots decreased.

In the current study, *WOX1* gene expression was abolished in the presence of the peptide AEDL. However, in the tobacco seedlings exposed to the other experimental conditions (NaCl alone or NaCl and AEDL), there was a 10–15% increase in the expression of this gene compared with that of the control seedlings. Expression of the *WOX5* gene was halved under all experimental conditions compared with that of the control. Decreases in the expression of the *WOX7* and *WOX11* genes were detected only when the peptide AEDL was present. In the combined presence of the peptide and NaCl, expression of the *WOX7* gene decreased more than in the presence of the peptide alone (almost 50% for AEDL and NaCl versus approximately 30% for AEDL alone). Thus, in all experimental conditions in the presence of AEDL, a decrease in the expression of the *WOX* genes was observed. This indicates that the peptide AEDL contributes to increasing the pool of stem cells and decreasing their differentiation.

## 3. Discussion

Roots are critical to plant development as they extract water and nutrients from the soil [1]. The ability of roots to absorb minerals and water from the soil and respond to environmental changes depends on the architecture and size of the root system. In the current study, the root lengths of the tobacco seedlings grown on medium containing AEDL were twice that of the control seedlings, and the height of the shoots of the experimental plants grown with AEDL were 20% higher than those of the control seedlings. These differences persisted under salinity conditions. Biometric data indicated the protective effect of AEDL on both root length and seedling height (Figure 1, Table 1).

Localization of the peptide in the roots was determined by fluorescence microscopy using FITC-labelled AEDL. A strong fluorescent signal was observed in the zones of elongation and root hair (Figure 2), while the fluorescence was insignificant in the meristem zone. Consequently, AEDL can not only penetrate the nuclei but can also accumulate within this organelle to a large extent in comparison with the cytoplasm. Thus, the detection and accumulation of AEDL in the root of tobacco seedlings is, to a certain extent, tissue-specific. This might be due to the cells in different root zones exhibiting differing competence for interaction, penetration, and accumulation of the peptide.

The packaging of genetic material into nucleosomes is a distinctive evolutionary feature of eukaryotic cells. The chromatin structure is very dynamic and can change during growth and development, as well as in response to environmental influences. The organization of DNA into chromatin is critical for proper temporal and spatial regulation of gene expression in most eukaryotic organisms.

Electron micrographs show the changes in the density of plant nuclei when grown with AEDL and under the influence of salinity. In the presence of AEDL and NaCl, nuclear chromatin was condensed to a greater extent than before salinization; the area of condensed chromatin in the nucleus increased compared with the area of diffused chromatin. The action of the peptide AEDL leads to a change in the structural organization of the nuclei with an increase in the size and number of clusters of condensed chromatin (Figure 3). Therefore, the presence of AEDL affects the structural organization of nuclei and chromatin morphology, which potentially cause significant changes in the expression of numerous genes associated with development and differentiation.

The intensity of ROS marker fluorescence in plant cells grown with the addition of AEDL was much lower both in the presence and absence of NaCl compared with the control, indicating the protective properties of the peptide under oxidative stress. Upon salinization with NaCl, ROS is known to perform both signaling and regulatory functions in plant cells [45]. NaCl is extremely toxic to plants, and the hyperosmotic stress, ion imbalance, and altered water status arising from NaCl treatment cause further inhibition of plant growth and molecular damage to cells during the formation of ROS [45]. It is believed that the effect of ROS as signaling mediators is determined not only by the number of ROS molecules but also by cell localization and interaction with antioxidants, other signaling molecules, and stress phytohormones [46]. Root elongation occurs either by increasing the process of stem cell differentiation in the meristem or by weakening the cell wall in the root EZ.

Meristems, which contain pools of stem cells, control the continuous growth and development of plants. The size of the meristem is directly related to the rate of root growth [47]. Division of stem cells and differentiation of their offspring are coordinated by intercellular signaling. Balancing the rate of proliferation and differentiation in the root is critical for root growth since root elongation is largely determined by the number of cell divisions of stem cell precursors and their subsequent cell expansion [47]. Multiple pathways/factors affect the regulation of the cell cycle, including ROS [37], DNA damage [48,49,50], and plant hormones [51].

The identity of stem cells is reportedly determined by signaling from a group of cells that organize a quiescent center (QC), in which the TF homeodomain WUS functions [52]. *WUS* expression is limited to a small group of cells in the QC [52,53]. Loss of WUS function leads to stem cell differentiation [53]. In contrast, *WUS* expression in the bud of vegetative organs induces the identity of ectopic stem cells [54], indicating that *WUS* expression must be tightly controlled to maintain the required number of stem cells. Stem cell homeostasis requires a dynamic feedback loop involving QC signaling and transport of the WUS protein [55], which maintains the pluripotency of stem cells and directly activates transcription of the CLV3 signal peptide gene, and this, in turn, represses *WUS* transcription through receptor signaling kinases [54,56,57,58].

The small, secreted CLV3 polypeptide acts in the extracellular space as a negative regulator of *WUS* expression [59,60]. CLV3 protein is involved in communication between stem cells and their neighbors. It has been demonstrated that CLV3 protein can spread from producing cells to the neighboring cells, where it suppresses WUS expression, and that this action on neighboring cells is necessary for stem cell homeostasis. Stem cells also indirectly inhibit their daughter cells laterally, allowing them to initiate differentiation. The gene CLV1 encodes a putative transmembrane receptor rich in leucine residues in the kinase domain, which is expressed at the SAM center [61]. Strong expression of CLV1 at the center of the meristem limits the movement of CLV3 away from stem cells. Thus, CLV1 protects the QC from the entry of CLV3 into it, which allows the expression of *WUS* in the QC and ensures the continued production of stem cells and the activity of the meristem. This regulated proliferation of the secreted CLV3 signal peptide allows the shoot meristem to determine the onset of cell differentiation in the periphery while simultaneously maintaining a stable niche for stem cells in the QC.

In the current study, expression of the *WOX* genes was abolished in the tobacco seedlings grown in the presence of the peptide AEDL, compared with the control seedlings. This was in accordance with the literature data and suggests a decline in the stem cell population and an increase in their differentiation [12]. The increase in stem cell differentiation in the *N. tabacum* grown in the presence of AEDL resulted in elongation of the main root of the plants compared with the control seedlings. The addition of 150 mM NaCl to the nutrient medium was accompanied by a slight increase in the expression of *WOX1, WOX7*, and *WOX11* genes, while *WOX5* expression decreased by almost 25%. In the presence of both AEDL and NaCl, there was a decrease in the expression of the *WOX7* and *WOX11* genes, which are responsible for the organogenesis of root cells [17,18]. It is assumed that the peptide AEDL acts like the CLV3 peptide (although they differ significantly in molecular weights). This assumption was supported by the fluorescence microscopy data showing that the FITC-labeled AEDL penetrated the tobacco root by the symplast through the plasmodesmata state [62] and was predominantly localized in the EZ and was rarely/insignificantly detected in the meristem zone. Thus, FITC-AEDL does not penetrate the QC zone and the stem cell niche. 

It is hypothesized that the peptide AEDL containing a hydrophobic leucine residue can bind to a hydrophobic leucine motif on the CLV1 receptor, which prevents AEDL from entering the stem cell niche [63]. The binding of the peptide AEDL to the CLV1 receptor leads to the activation of the receptor complex, thereby limiting the stem cell population and activating the differentiation of these stem cells. Concurrently, the formation of a complex between the peptide AEDL and the CLV1 receptor prevents the CLV3 peptide from entering the meristem from the QC zone. Overexpression of the CLE40 peptide leads to disorganization of the tissue layer and premature differentiation of root stem cells [64]. CLE40 can completely replace CLV3 for activating CLV signaling in the shoot, indicating that CLV3 and CLE40 are functionally equivalent proteins that differ mainly in the character of expression [64].

Plant cell walls expand or relax as a consequence of the molecular creep process, in which cellulose microfibrils and associated matrix polysaccharides are separated from each other [65]. The energy required to overcome the viscous resistance and entanglement of the wall polymers comes from the cell-wall stress, which arises from the turgor pressure inside the cells of living plants. Such molecular creep occurs only when the cell wall is loosened by expansins or other factors [28]. A non-enzymatic mechanism of action of expansins has been proposed, in which expansins destroy the non-covalent bonds that bind the matrix polysaccharides to the surface of cellulose microfibrils or each other [66,67,68]. Expansin activity is frequently associated with the weakening of the cell wall in growing cells [69]. In most cases, suppression of expansin genes results in growth inhibition, while excessive ectopic expression leads to faster or abnormal growth. The localized expression of expansins is associated with the meristems and growth zones of plant roots and stems, as well as with the formation of leaf primordia on the apical meristems of the shoot [70] and with the proliferation of epidermal cell walls during root-hair formation [71]. The expression of specific expansin genes is induced by hormones, stresses, or other stimuli [72].

In *N. tabacum*, the expression of the expansin genes *EXPA3* and *EXPA5* were similar in the control plants and in those exposed to the peptide AEDL. Thus, this suggests that the lengthening of the roots in the presence of the peptide does not occur due to the weakening of the cell wall in the EZ. A 50% decrease in *EXPA5* expression in the presence of a high concentration of NaCl was accompanied by a decrease in root length and root bending. However, in tobacco plants grown in the presence of NaCl and AEDL, there was a significant increase in the expression of both *EXPA3* and *EXPA5* (more than threefold versus the control). This increase in expression is probably due to the presence of NaCl inducing a change in the structure of chromatin—the chromatin became more decondensed and accessible for the penetration of the peptide AEDL and the subsequent regulation of expansin expression. Consequently, an increase in the activity of the expansin genes contributes to the weakening of the cell wall, especially in the EZ, and thus results in the elongation of the plant roots.

## 4. Materials and Methods

### 4.1. Object of Study

Seeds of tobacco (*Nicotiana tabacum* L.) cultivar Samsun (collection of Agricultural Academy, Moscow, Russia) were placed in flasks (150 mL) containing hormone-free Murashige–Skoog (MS) agar medium supplemented with or without additives: (1) control; (2) 10^−7^ M AEDL; (3)150 mM NaCl; (4)150 mM NaCl and 10^−7^ M AEDL. Glass flasks were placed in a climatic chamber (Fisons, Ipswich, UK) [72]. Cultivation was carried out at 24 °C with artificial lighting with daylight lamps (5000 lux) day/night—14/10 h, respectively. After 28 days of cultivation, the length of the main root and the height of the shoot were measured. Experiments were carried out in four replicates. The parameters were measured using an Olympus BX51 microscope (Olympus, Tokyo, Japan) with the Cell program (Soft Imaging System, Münster, Germany).

### 4.2. Preparation of FITC-Labeled AEDL

A solution of FITC (Sigma-Aldrich, St. Louis, MO, USA) (1 μg in 10 μL of 0.5 M sodium bicarbonate) was added to a solution of AEDL in 0.01 M Tris-HCl buffer (pH 7.0) at a ratio of 1.2:1 [73]. The reaction mixture was incubated at room temperature for 30 min with constant shaking. The obtained fluorescently labeled peptide AEDL were analyzed by chromatography on a BioLogic DuoFlow chromatograph (BioRad, Hercules, CA, USA) on a C-18 column (20 cm) in a concentration gradient of acetonitrile (0–100%) (Panreac, Barcelona, Spain) containing 1% trifluoroacetic acid.

### 4.3. Fluorescence Microscopy

To determine the localization of peptides in root cells, the FITC-labeled AEDL was used at a concentration of 10^−5^ M; control FITC (10^−5^ M) without peptides was used as controls [72]. The incubation time was 20 h.

The root tips (4–5 mm) were separated and placed on a glass slide in a drop of distilled water. To image the ROS in intravital cells, we used an aqueous solution of 5-(and-6)-carboxy-2′,7′-difluorodihydrofluorescein diacetate (Carboxy-H_2_DFFDA) (Thermo Fisher Scientific, Waltham, MA, USA) at a concentration of 25–50 nM; the incubation time was 30 min, followed by three washes in distilled water. Fluorescence was analyzed at a wavelength of 490 nm using a BX51 microscope (Olympus, Tokyo, Japan) with 10× and 20× objective lens. Photographs were obtained using a Color Vien II digital camera with the Cell program (Soft Imaging System, Münster, Germany).

### 4.4. Transmission Electron Microscopy (TEM)

Samples of the root tip (4 mm) were fixed for 4 h at room temperature in a 2.5% solution of glutaraldehyde in 0.1 M Na-phosphate buffer, pH 7.2, containing sucrose (15 mg/mL). After washing in the same buffer, probes were post-fixed using 1% OsO_4_ (Sigma, St. Louis, MO, USA) on ice for 2 h. Later, the material was dehydrated and embedded into Epoxide resin (Epon-812 and Araldite mixture, Merck, Darmstadt, Germany) using a standard technique [73]. Ultrathin sections were prepared with an LKB-III microtome (LKB, Stockholm, Sweden). The ultra-thin (1–2 μ) sections were contrasted with lead citrate and viewed using an H-500 transmission electron microscope (Hitachi, Tokyo, Japan) at an accelerating voltage of 10 kV and operating magnification of 10,000×. For chromatin morphology statistical analyses, we measured the area of the nucleus using the Cell program. Its darkly contrasted and electron-transparent chromatin areas correspond to the dense and loose chromatin, respectively. We used more than 30 photos with cell 60 nuclei of the interphase nucleus of meristem of progeny stem cells. The parameters were measured using a BX51 microscope (Olympus, Tokyo, Japan) furnished with the Cell program.

### 4.5. RNA Isolation

Total RNA was isolated from the tobacco roots using the RNA-Extran RNA isolation reagent kit (Syntol, Moscow, Russia) according to the manufacturer’s instructions. The concentration of isolated RNA preparations was determined on a NanoPhotometer IMPLEN (Westlake Village, CA, USA).

Then, the cDNA was obtained using a set of reverse transcription reagents (Syntol, Moscow, Russia) according to the manufacturer’s instructions.

### 4.6. Real-Time PCR

Information on the primary structure of *Nicotiana tabacum* genes was obtained from the National Center for Biotechnology Information (NCBI). Primers for these genes were designed using the NCBI Primer-BLAST web tool and synthesized at Syntol (Table S2). Information about primers is presented in the Supplementary Materials. Real-time PCR was performed on CFX96 Touch Real-Time PCR Detection System (Bio-Rad) using a set of RT-PCR reagents and SYBR Green (Syntol). The PCR-RT reaction was carried out under identical conditions for all samples: 95 °C—5 min, then 45 cycles—94 °C—30 s, 58 °C—30 s, 72 °C—30 s. The reaction was carried out in 2–3 parallels and in three repetitions. The relative expression level of genes was calculated using a calibration curve constructed with PCR products derived from the *GAPDH* gene. The analysis of relative gene expression data was performed using the 2^−ΔΔCt^ method [74].

### 4.7. Statistical Processing of Results

The calculation of the main statistical parameters was carried out according to standard methods, and Statistica 6.0 and STATAN programs for statistical data processing were used. Values are presented as means ± standard deviation of the three biological replicates. All of the treatment effects were statistically analyzed using the Student’s *t*-test (DPS software, Newcastle, UK). Different letters indicate significant differences at *p* < 0.05.

The equipment of the Center for Collective Use of the Federal State Budgetary Scientific Institution VNIISB RAS was used in this study.

## 5. Conclusions

It has been shown that the peptide AEDL has a lengthening effect on the root cells of tobacco seedlings, and the effect of this peptide at such a low concentration is similar to the effect of peptide phytohormones. With the combined presence of AEDL and NaCl, root development is normalized in comparison with the action of NaCl alone. It was found that the localization and accumulation of AEDL at the root are tissue-specific. The localization of peptides can be effectively used to quickly assess the state of root cells of plants grown under various conditions, including under the influence of various factors.

It was revealed that the action of the peptide AEDL leads to a change in the structural organization of chromatin. An increase in chromatin condensation in the presence of AEDL leads to significant changes in the expression of various genes associated with the development and differentiation of the root system.

It was found that the elongation of the roots of tobacco seedlings in the presence of the peptide AEDL and in the combined presence of AEDL and NaCl occurs by different mechanisms.

Based on the obtained results, it can be assumed that the peptide AEDL can be used to increase plant tolerance to abiotic stresses, including high concentrations of sodium chloride.

## Figures and Tables

**Table 1:** The height and length of tobacco seedlings grown under different conditions.

Variant	Shoot Height, mm	Root Length, mm
Control	7.2 ± 2.3	10.2 ± 2.1
+AEDL	9.1 ± 3.1	21.2 ± 3.1
+150 mM NaCl	7.3 ± 1.2	12.7 ± 2.4
+ AEDL +		
150 mM NaCl	4.2 ± 1.5	7.1 ± 2.2

Mean ± standard error (*n* = 30). Penetration of FITC-AEDL into tobacco roots.

## Data Availability

Not applicable.

## Supplementary Materials

The following supporting information can be downloaded at: https://www.mdpi.com/article/10.3390/plants11101352/s1, Table S1. Condensed and decondensed chromatin content in the nuclei of tobacco root cells, Table S2. Primers for RT-PCR.
