# Supplementary material for: Elongating Effect of the Peptide AEDL on the Root of Nicotiana tabacum under Salinity
Source: Plants (Basel). 2022 May 19;11(10):1352. doi: 10.3390/plants11101352 (PMC9147445; doi:10.3390/plants11101352)
Supplement: Supplementary file 1 [file plants-11-01352-s001.zip › plants-1711501-supplementary.pdf]

Table S1. Condensed and decondensed chromatin content in the nuclei of tobacco root cells

**Treatment**	**Condensed chromatin,%**	**Decondensed chromatin,%**
**Control**	**42,3±1,2**	**57,7±1,3**
**+AEDL**	**69,6±2,0**	**30,4±1,1**
**+150 mM NaCl**	**64,3±2,8**	**35,7±1,8**
**+AEDL+ 150 mM NaCl**	**30,2±1,6**	**69,8±2,3**

Table S2. Primers for RT-PCR

**gene**	**5′-3′-sequence**
EXPA3	TGT CCA AAG TTG GTG TAA CAG GA<br>TGA AGA TTG CAG CTG AGG CA
EXPA5	GGC TTT ATT GCT GTG GTG GC<br>AAG TCG CCC CAA TTC TGC TT
WOX1	CAC CCA TTT TAG TTA TTC TCC CCC<br>TGT TGA AAT CTC CCC CGT CG
WOX5	TCA AGG GTC CGAACA ACC AG<br>CAA ACT TTC AGC CCC ACC TG
WOX7	TGT TCA GGT CAG GAC TCC GT<br>ACT CTG CGA CGT TTC TGT CT
WOX11	TTG CCA AAT TCC TTG TCT TCA GC<br>CCC GTC GTT GTA ACC CAT CA
